# Increased Force Variability in Chronic Stroke: Contributions of Force Modulation below 1 Hz

**DOI:** 10.1371/journal.pone.0083468

**Published:** 2013-12-26

**Authors:** Neha Lodha, Gaurav Misra, Stephen A. Coombes, Evangelos A. Christou, James H. Cauraugh

**Affiliations:** 1 Department of Applied Physiology and Kinesiology, Malcom Randall VA Medical Center, University of Florida, Gainesville, Florida, United States of America; 2 Department of Physical Therapy, Malcom Randall VA Medical Center, University of Florida, Gainesville, Florida, United States of America; 3 Brain Rehabilitation Research Center of Excellence, Malcom Randall VA Medical Center, University of Florida, Gainesville, Florida, United States of America; Centre Hospitalier Universitaire Vaudois Lausanne - CHUV, UNIL, Switzerland

## Abstract

Increased force variability constitutes a hallmark of arm disabilities following stroke. Force variability is related to the modulation of force below 1 Hz in healthy young and older adults. However, whether the increased force variability observed post stroke is related to the modulation of force below 1 Hz remains unknown. Thus, the purpose of this study was to compare force modulation below 1 Hz in chronic stroke and age-matched healthy individuals. Both stroke and control individuals (*N* = 26) performed an isometric grip task to submaximal force levels. Coefficient of variation quantified force variability, and power spectrum density of force quantified force modulation below 1 Hz with a high resolution (0.07 Hz). Analyses indicated that force variability was greater for the stroke group compared with to healthy controls and for the paretic hand compared with the non-paretic hand. Force modulation below 1 Hz differentiated the stroke individuals and healthy controls, as well as the paretic and non-paretic hands. Specifically, stroke individuals (paretic hand) exhibited greater power ∼0.2 Hz (0.07–0.35 Hz) and lesser power ∼0.6 Hz (0.49–0.77 Hz) compared to healthy controls (non-dominant hand). Similarly, the paretic hand exhibited greater power ∼0.2 Hz, and lesser power ∼0.6 Hz than the non-paretic hand. Moreover, variability of force was strongly predicted from the modulation of specific frequencies below 1 Hz (*R* ^2^ = 0.80). Together, these findings indicate that the modulation of force below 1 Hz provides significant insight into changes in motor control after stroke.

## Introduction

Stroke often affects motor control of the upper limb. A major impairment following stroke is the inability to control force, which persists even after extensive rehabilitation. Therefore, impairments in force control constitute a hallmark of arm disabilities following stroke. Our recent findings suggest that impaired force control in healthy older adults is related to a differential modulation of force below 1 Hz [1]. The focus of this study was to determine whether force control impairments in stroke individuals are related to a differential modulation of force below 1 Hz.

Impaired force control is often demonstrated as increased force variability [2]–[4]. When stroke individuals attempt to maintain a constant force, their variability is ∼45% higher than healthy age-matched controls [5]–[8]. This finding is consistent across various force levels ranging from 5–50% of maximum. Further, stroke patients with greater severity of stroke (lower Fugl-Meyer scale score) exhibit greater force variability [6]. Therefore, force variability is a functionally relevant index of motor performance because of the strong association with the severity of stroke impairment.

Force variability is characterized by oscillations in specific frequency bins. Earlier studies suggest that the greatest power in force oscillations occur below 4 Hz [9]–[11]. However, our recent findings demonstrate that the modulation of force occurs primarily below 1 Hz in young and older adults [1]. Changes at specific frequencies within this range may reflect changes in basic motor physiology and visuomotor processing. Indeed, previous observations have shown that an age-associated modulation of force can be split into two sub peaks [1]. The first sub peak occurs ∼0.2 Hz and is positively associated with force variability, whereas the second sub peak occurs ∼0.6 Hz and is negatively associated with force variability. Stroke individuals exhibit altered activation of motor units [12]–[14] and impaired respiratory rhythm [15], which leads to the suggestion that stroke may also be associated with changes in force production at very low frequencies. Furthermore, there is evidence that stroke individuals exhibit difficulty in processing visual information [16], and a peak ∼0.6 Hz has been associated with visuomotor processing [1]. Specific force oscillations below 1 Hz, may therefore have important implications for understanding the increased force variability and consequently pathological and dysfunctional motor control following stroke.

In this study, we investigated whether the increased force variability in stroke individuals was related to an altered modulation of force below 1 Hz. To examine this, we asked stroke and age-matched healthy participants to perform constant isometric contractions with their hands at different force levels. We hypothesized that stroke individuals would exhibit differential modulation of force below 1 Hz and that this modulation would be related to increased force variability.

## Methods

### Ethics Statement

The University of Florida’s Institutional Review Board approved the procedures involved in this study. All individuals read and signed the informed consent prior to participation.

### Participants

Chronic stroke (*N* = 13, age = 65.64±7.35 years; time post stroke = 4.75±4.04 years) and age-matched controls (*N* = 13; age = 65.59±9.40 years) volunteered to participate in this study. Clinical characteristics of the stroke individuals are shown in Table 1.

**Table 1:** Demographics of the Stroke Group.

Subject	Gender	Age(yr)	Duration(yr)	DominantLimb^a^	StrokeType	Affectedhemisphere	LesionLocation	FMA^b^	MAS^c^
1	F	69.33	5.08	R	I	R	Cortical	61	0
2	M	63.3	3.92	R	I	L	Sub-cortical	59	0
3	M	77.17	1.5	R	I	L	NA	55	0
4	M	60.13	1.75	R	I	L	Sub-cortical	50	1
5	M	70.33	3.5	L	I	L	Cortical	51	1
6	F	59.58	1.67	R	I	R	Cortical	49	1
7	M	58.83	0.75	R	I	L	Sub-cortical	44	1
8	M	63.92	11.75	L	H	L	Sub-cortical	34	3
9	M	69.93	3.08	L	H	L	Cortical	32	3
10	M	77.33	9.75	R	I	R	Cortical	30	2
11	M	56.08	1.17	R	I	L	NA	20	3
12	M	56.5	12.42	R	I	L	NA	16	3
13	F	71.00	5.42	R	H	L	Cortical	14	3

*Abbreviations: M-Male, F-Female, L-Left, R-Right, I-Ischemic, H-Hemorrhagic, NA-Not available.*

^a^ *Dominant Limb indicates premorbid handedness.*

b *Fugl-Meyer Assessment Score for impaired upper extremity (0–66). Participants with higher FMA scores have less motor impairments.*

c *Modified Ashworth Scale (0–4) of the wrist determined the presence of hypertonia in the hand and wrist muscles, such that higher scores indicate increased muscle tone.*

Inclusion criteria for stroke participants follow: (1) diagnosed with a single unilateral cerebrovascular accident at least 9 months prior to testing; (2) ability to voluntarily open and close fingers in a fist on command; and (3) a minimum of 10° of wrist and finger extension without assistance; (4) intact cognition (Mini Mental State Examination score >23 [17]. Exclusion criteria included the presence of any other neurological or musculoskeletal deficit, uncorrected vision, hearing impairments, and pain in upper extremity or elsewhere that could interfere with the hand movements. Self-report on the above impairments as well as visual neglect was used to screen participants.

### Procedure

#### Clinical evaluations

Hand and arm motor impairments of the stroke participants were assessed using the upper extremity subsection of the Fugl-Meyer Motor Assessment (FMA) [18], [19]. Muscle tone in the hands was evaluated using the Modified Ashworth Scale (MAS) [20]. Handedness for the control group was evaluated on the Edinburgh Handedness inventory [21]. Stroke participants’ self-reported measures of premorbid hand dominance were recorded.

#### Participant Positioning

**Figure 1:**
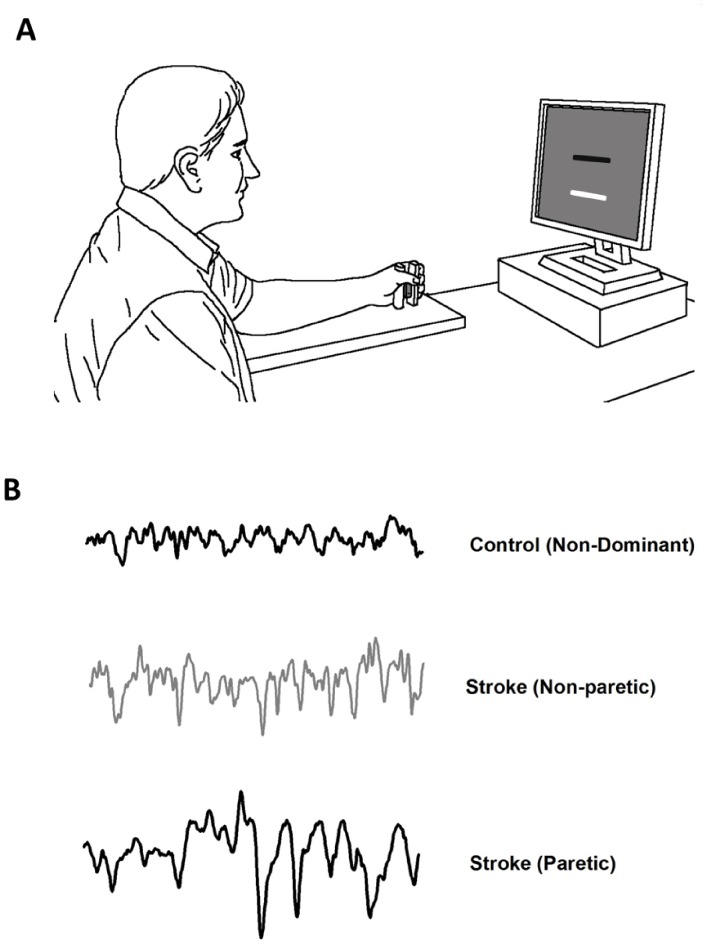
The experimental setup. *(A) The arm and hand positions on the gripping apparatus.* Participants executed unimanual isometric grip to match their forces (white bar) as accurately as possible to the target force (black bar) at 5%, 25% and 50% of MVC. (B) Typical force trials of an age-matched control and stroke participant at 25% submaximal target force for 20 s.

Figure 1A shows the experimental set-up with the position of the arm and hand on the gripping apparatus during an isometric contraction.

Participants sat in a comfortable chair with their forearms and elbows supported on a 70 cm high table positioned directly in front of their chair. Participants maintained a straight trunk with approximately 15–20° of shoulder flexion, 20–45° of elbow flexion, and 10–15° of wrist extension. A custom-designed gripping apparatus embedded with a force transducer was firmly attached to a platform to prevent forward/backward sliding, sideways tilting, or twisting by the participants during task execution. The gripping apparatus was positioned 15–25 cm from the body midline. Participants performed power grip by flexing the digits and holding the apparatus between the fingers and thumb. No physical restraint was used to avoid augmenting any sensory input during trials.

The participants were instructed to sit up straight and keep their elbow resting on the surface at all times during a trial. They were asked to begin gripping the apparatus at the trial onset and maintain a stable posture until trial completion. A physical therapist carefully monitored postures throughout the experiment. Extraneous movements such as pushing with the elbows, moving the shoulders, leaning forward were discouraged. Additionally, participants failed to comply with instructions on only 16 of 468 trials, and these trials were eliminated from analyses.

#### Maximum Voluntary Contraction (MVC)

Each participant’s maximal isometric force was calculated for both hands. Participants were instructed to apply as much force as possible on hearing the ‘go’ beep and hold the force until they heard the second ‘end’ beep. Online visual feedback was given to motivate performance. For each hand, participants completed 3 trials of 6 s duration with a rest period of 90 s between trials. The mean of the 10 highest force samples computed from each trial was averaged to determine MVC. The order of the blocks (left hand, right hand) was randomized across participants.

#### Submaximal isometric force production

Three target force levels were determined as a percentage of the maximal force (5%, 25%, and 50% of MVC) produced by the hand. To become familiar with the task, participants practiced two trials at each force level before testing. At trial onset, participants executed a unimanual isometric grip to match a white moveable target bar as accurately as possible to the stationary black target bar on the computer monitor (Figure 1A). A submaximal force control trial lasted for 20 s. For each hand, three trials were administered at each unique force level condition. A total of 18 trials were completed with a rest period of 60****s between trials. The order of force levels (5%, 25%, 50%) within a block and block order (left hand, right hand) was randomized across participants.

#### Data acquisition

The load cells (MLP-200, 4.16×1.27×1.90 cm, range, 0.1% sensitivity, Transducer Techniques) were used to collect force data during MVC and submaximal force control task. The amplified (15LT Grass Technologies Physio-data Amplifier System, 10 V excitation voltage, 200 gain) force output was sampled (16-bit A/D convertor; NI cDAQ-9172+ NI-9215, National Instruments) at a rate of 100 Hz. A LCD computer monitor (43.2 cm screen, 1024×768 resolution, 100 Hz refresh rate) positioned at eye level was used to present trials. Participants viewed a stationary horizontal black bar (256×20 pixels) representing the target force, and a movable horizontal white bar (256×20 pixels) displaceable in the vertical direction, representing the isometric force produced by the participant in real time. A custom LabVIEW routine (8.1; National Instruments) controlled the visual presentation in each trial. Force data were saved for offline analysis.

#### Data analysis

The first 5 s and final 1 s of force data were eliminated from all analyses to account for early and late force adjustments. The 14 s force signal was filtered using a fourth-order Butterworth filter at a cut off frequency of 20 Hz. To eliminate any drift in force within a trial, the force data were linearly detrended. Motor performance was characterized by the variability and power spectrum of force output. Figure 1B shows representative force trials from a control and a stroke participant.

#### Coefficient of variation of force

To assess the magnitude of force variability within each trial, standard deviation (SD) was calculated by measuring the fluctuations around the mean force produced by the participants. Further, force variability was normalized to the magnitude of force, by computing coefficient of variation (CV), as CV of force = SD of force/mean force output×100.

#### Power spectrum of force

Time-series analysis using the Welch algorithm was performed to compute the power spectrum density of the force signal. The window size was 1400, which gave a resolution of 0.07 Hz. For statistical comparisons, the power spectrum of the force signal within 0–1 Hz was divided into fifteen frequency bins: 0–0.07, 0.07–0.14, 0.14–0.21, 0.21–0.28, 0.28–0.35, 0.35–0.42, 0.42–0.49, 0.49–0.56, 0.56–0.63, 0.63–0.70, 0.70–0.77, 0.77–0.84, 0.84–0.91, and 0.91–0.98 Hz. These frequency bins were based on the highest resolution that could be accomplished with 14 s of force data. The high resolution power spectrum allowed us to identify precisely the frequency bands below 1 Hz where greatest differences existed between the stroke and control groups. The dependent variables for the spectral analysis of the force signal were the absolute (N^2^) and normalized power (%) in each data bin. Normalized power was calculated as the absolute power in each frequency bin relative to the total power of the force signal from 0–1 Hz. In addition, we examined the power spectrum of force from 0–12 Hz to determine if the majority of power was located below 1 Hz.

### Statistical Analysis

To determine the influence of stroke on force variability, we analyzed the CV of force for the stroke (paretic hand) and control (non-dominant hand) participants using a 2 Group (stroke and control)×3 Force Level (5%, 25%, and 50% of MVC) mixed model analysis of variance (ANOVA) with repeated measures on force level. To examine the differences in the CV of force between the paretic and non-paretic hand of stroke participants, we used a 2 Hand (paretic and non-paretic)×3 Force Level (5%, 25%, and 50% of MVC) ANOVA with repeated measures on both factors.

To determine the modulation of normalized power below 1 Hz for stroke and control participants, we performed the following analyses. First, between groups comparisons were made using a 2×3×15 (Group×Force Level×Frequency) mixed design ANOVA with repeated measures on force level and frequency. Second, within group comparisons were made using a 2×3×15 (Hand×Force Level×Frequency) ANOVA with repeated measures on all factors. For all ANOVAs, when assumptions of sphericity were violated, Greenhouse-Geisser’s degrees of freedom adjustment were used. Significant interactions were followed by the Bonferroni’s post hoc procedure for mean comparisons.

We used a backward- stepwise multiple linear regression model to establish a statistical model that predicted the CV of force (dependent variable; criterion) from the absolute power of force below 1 Hz (independent variables; predictors). In addition, within the stroke group, we used a similar regression model to establish a statistical model that predicted the difference in CV of force between the paretic and non-paretic hand (dependent variable) from the difference in the absolute power of force below 1 Hz between hands (independent variables). The squared multiple correlation (*R^2^*) and the adjusted squared multiple correlation (adjusted *R^2^*) determined the goodness-of-fit of the model. *R^2^* indicates the robustness of the linear combination of the variables predicting the CV of force or the change in the CV of force. The adjusted *R^2^* accounts for *R^2^* ’s overestimate of the percentage of the variance in the criterion variable that can be explained by the linear combination of the predictor variables, especially when the sample size is small and the number of predictors is large. The backward model was accepted if all predictor variables significantly contributed to the criterion variable. In addition, we chose a model with the least number of predictors that demonstrated a significant adjusted *R^2^*. All statistical tests were conducted with alpha level set at 0.05. Only significant findings are reported in the Results. Analyses were performed with the IBM SPSS Statistics 21.0 statistical package.

## Results

### Strength

For the stroke individuals, the strength of the paretic hand was significantly reduced compared with the non-paretic hand (|*t*_12_| = −4.24; *p*<0.01). The MVC force for the paretic hand was 173.83 (±31.20) N, whereas the MVC for the non-paretic hand was 285.05 (±22.07) N. For the age-matched controls, no significant difference (|*t*_12_| = −0.06; *p*>0.05) was found between the dominant (297.83±22.4 N) and non-dominant hand (297.10±22.34 N).

To ensure that fatigue did not affect task performance, we computed root mean square error (RMSE) as the squared distance between target force and force produced. A paired sample *t-*test on RMSE confirmed that no difference existed between the first and the third trial at each force level for each group (all *p*’s >0.05).

### Force Variability

**Figure 2:**
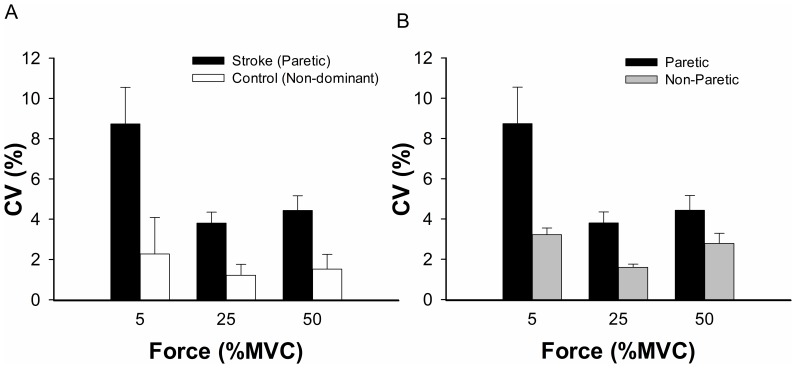
The coefficient of variation (CV) at the three force levels. (A) Differences in CV of force for stroke (paretic hand) and control (non-dominant hand) participants. The stroke participants demonstrated greater variability than the control participants (*p*<0.05). (B) Differences in CV of force for the paretic and non-paretic arm of the stroke participants. The paretic arm exhibited more variability than the non-paretic arm participants (*p*<0.05). Error bars represent standard error.

The CV of force for the paretic hand was greater than the non-dominant hand of controls (Figure 2A; Group main effect: *F*_1,24_ = 10.78, *p*<0.05, *η^2^* = 0.31). Within the stroke group, the CV of force for the paretic hand was greater than the non-paretic hand (Figure 2B; Hand main effect: *F*_1,14.82_ = 6.20, *p*<0.05, *η^2^* = 0.34). Therefore, the paretic hand exhibited greater force variability than the non-dominant hand of control participants and the non-paretic hand of stroke participants.

### Power Spectrum of Force

**Figure 3:**
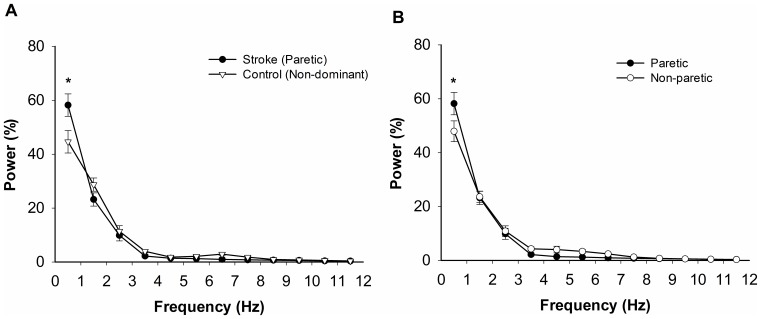
Normalized power spectrum of the force output from 0–12 Hz. The majority of the power was concentrated below 1

Figure 3 shows the averaged normalized power spectrum of force from 0–12 Hz. Indeed, Figure 3 demonstrates that the majority of the power was located below 1 Hz for all hands tested. In addition, the greatest difference between the paretic hand and non-dominant hand of the control individuals was below 1 Hz (13.59±5.884%). Similarly, the greatest difference between the paretic hand and non-paretic hand (10.28±3.59%) was below 1 Hz.

**Figure 4:**
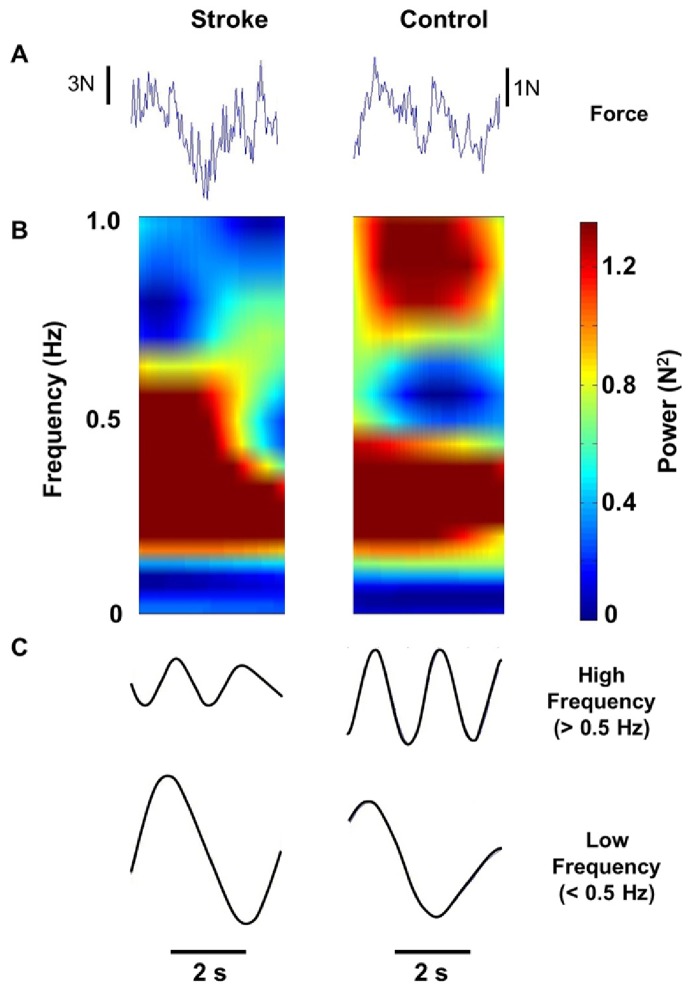
Representative data for a stroke and a control participant. (A) Forces (duration = 2 s). (B) Power spectrum below 1 Hz (Y axis) across time (X axis). The warmer colors (i.e., red) indicate greater power and cool colors (i.e., blue) indicate reduced power. On the left side, the stroke participant demonstrates high power in 0–0.5 Hz and low power in 0.5–1 Hz. In contrast, the control participant (on the right side) demonstrates high power in both 0–0.5 Hz and 0.5–1 Hz. (C) The top panel shows the force band-passed between 0.5–1 Hz. At higher frequencies (>0.5 Hz), the control participant exhibits higher power (amplitude) than the stroke participant. The bottom panel shows the force output low pass filtered at 0.5 Hz. At lower frequencies (<0.5 Hz), the stroke participant exhibits greater power (amplitude) than the control participant.

Figure 4 shows a typical example of the modulation of force below 1 Hz. The stroke participant exhibited higher CV of force than the control participant (Figure 4A). The wavelet of force (Figure 4B) demonstrates that the stroke and control participants exhibited differential modulation of spectral power below 1 Hz. Specifically, the stroke participant demonstrated increased power in low frequencies (<0.5 Hz; Figure 4C) and reduced power at higher frequencies (>0.5 Hz) of force.

### Modulation of Power Spectrum of Force Below 1 Hz

**Figure 5:**
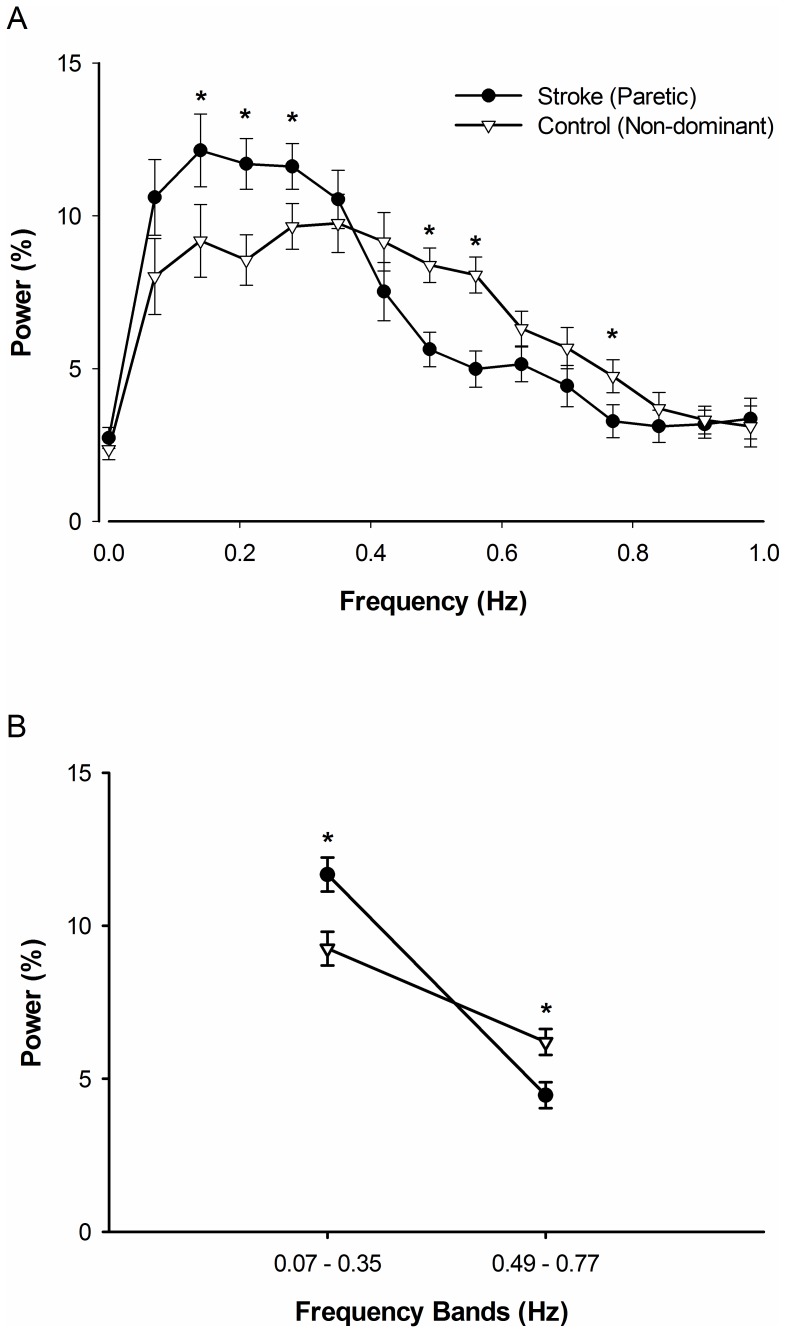
The modulation of power below 1 Hz for stroke and control groups. *(A)* The stroke group demonstrated significantly greater relative power at 0.14, 0.21 and 0.28 Hz compared with the control group (*p*<0.05). However, this relationship reversed at higher frequencies. Specifically, the stroke group had significantly reduced power at 0.49 Hz, 0.56, and 0.77 Hz compared with the control group (*p*<0.05). (*B)* The average power in frequency bands 0.07–0.35 Hz and 0.49–0.77 Hz for stroke and control participants. The stroke group shows greater power within 0.07–0.35 Hz (*p*<0.05) and reduced power within 0.49–0.77 Hz (*p*<0.05).

The interaction between group and frequency for the normalized power spectrum of force below 1 Hz was significant (*F*_4.47, 186.24_ = 3.14, *p*<0.05, *η^2^* = 0.12; Figure 5A). Visual inspection of the interaction indicated that the paretic hand exhibited greater normalized power from 0.1 to 0.3 Hz and lesser normalized power from 0.5 to 0.8 Hz. Post-hoc analysis revealed that the paretic hand demonstrated increased power at 0.14 Hz (|*t*_24_| = 1.76, *p*<0.05), 0.21 Hz (|*t*_24_| = 2.67, *p*<0.05), and 0.28 Hz (|*t*_24_| = 1.86, *p*<0.05) and reduced power at 0.49 Hz (|*t*_24_| = −3.45, *p*<0.05), 0.56 Hz (|*t*_24_| = −3.67, *p*<0.05), and 0.77 Hz (|*t*_24_| = −1.93, *p*<0.05) compared with the non-dominant hand in control participants. Based on this finding, we divided the force spectrum below 1 Hz into two broader frequency bands 0.07 to 0.35 and 0.49 to 0.77 Hz. Further analysis indicated that the interaction between group (2) and frequency bands (2) for the normalized power spectrum of force was significant (*F*_1, 24_ = 11.17, *p*<0.005, *η^2^* = 0.32; Figure 5B). Post-hoc analysis revealed that the paretic hand demonstrates increased power within 0.07 to 0.35 Hz (|*t*_24_| = 3.22, *p*<0.005) and reduced power within 0.49 to 0.77 Hz (|*t*_24_| = −3.17, *p*<0.005) compared with the non-dominant hand in control participants.

**Figure 6:**
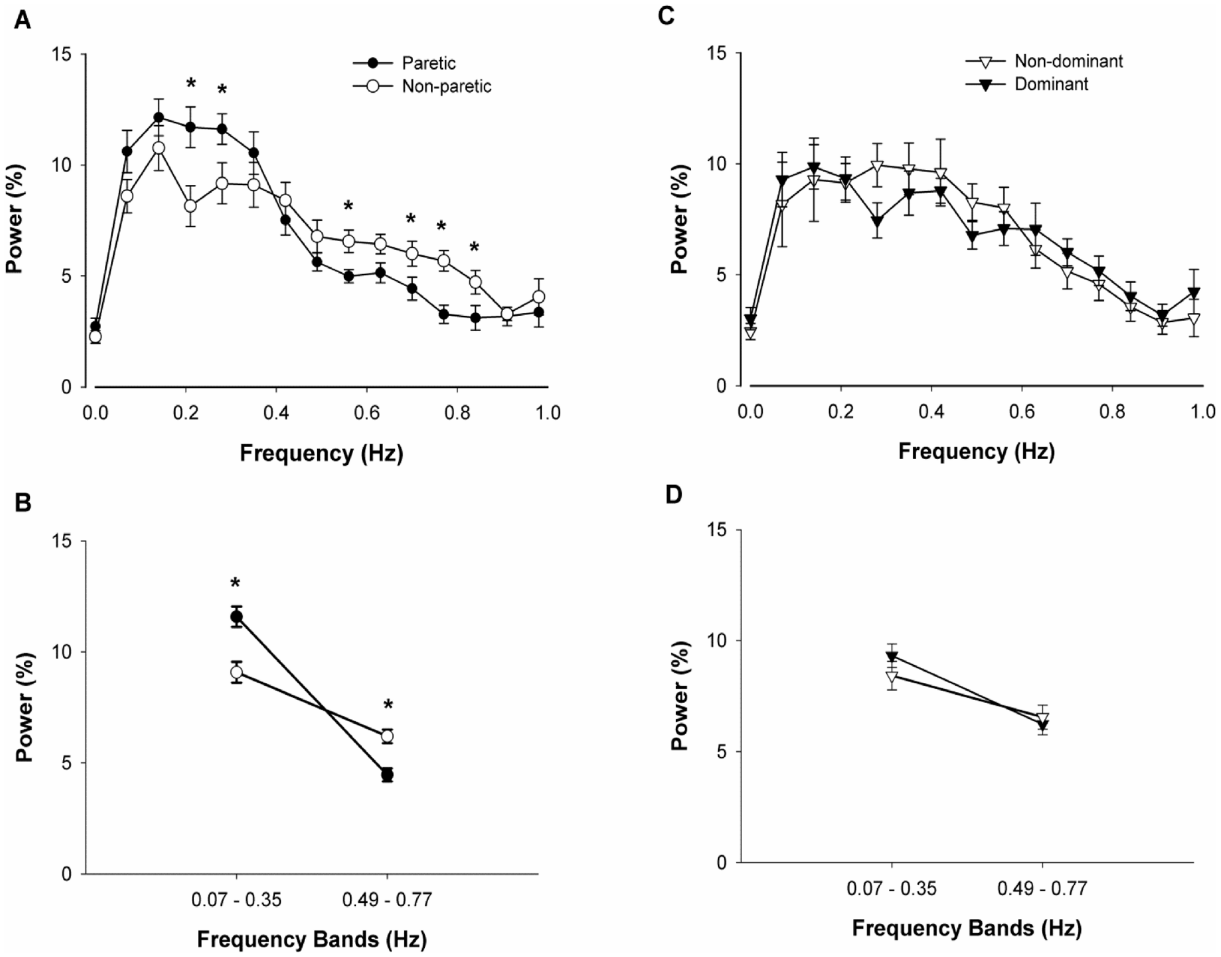
The modulation of power below 1 Hz within stroke and control groups. (A) For the stroke group, the paretic hand demonstrated significantly greater power at 0.21 and 0.28 Hz relative to the non-paretic hand (*p*<0.05). Further, the paretic hand showed significantly reduced power at 0.56 Hz, 0.7 Hz, 0.77 Hz and 0.84 Hz compared with the non-paretic hand (*p*<0.05). (B) The power in frequency bands 0.07–0.35 Hz and 0.49–0.77 Hz within stroke group**.** For the stroke group, the paretic hand shows greater power within 0.07–0.35 Hz (*p*<0.05) and reduced power within 0.49–0.77 Hz (*p*<0.05) relative to the non-paretic hand. (C) For the control group, the dominant hand’s power density below 1 Hz did not vary significantly from the non-dominant hand. (D) Similarly, the power in frequency bands 0.07–0.35 Hz and 0.49–0.77 Hz does not differ between the dominant and the non-dominant hand for the control group.

Additional analysis of the stroke group on the normalized power spectrum of force below 1 Hz revealed a significant Hand×Frequency interaction (*F*_14, 168_ = 2.99, *p*<0.01, *η^2^* = 0.20; Figure 6A). Visual inspection of the interaction indicated that the paretic hand exhibited increased normalized power from 0.1 to 0.3 Hz and reduced normalized power from 0.5 to 0.8 Hz. Follow-up tests revealed that the paretic hand exhibited increased power at 0.21 Hz (|*t*_12_| = 3.21, *p*<0.05) and 0.28 Hz (|*t*_12_| = 2.09, *p*<0.05) and reduced power at 0.56 Hz (|*t*_12_| = −2.37, *p*<0.05), 0.7 Hz (|*t*_12_| = −1.98, *p*<0.05), 0.77 Hz (|*t*_12_| = −4.02, *p*<0.05) and 0.84 Hz (|*t*_12_| = −2.56, *p*<0.05) compared with the non-paretic hand. Similar to the above analysis, we divided the force spectrum below 1 Hz into two broader frequency bands 0.07 to 0.35 and 0.49 to 0.77 Hz. The interaction between hand (2) and frequency bands (2) for the normalized power spectrum of force was significant (*F*_1, 12_ = 14.24, *p*<0.005, *η^2^* = 0.93; Figure 6B). Follow-up tests indicated that the paretic hand showed increased power at 0.07 to 0.35 Hz (|*t*_12_| = 3.32, *p*<0.05) and reduced power at 0.49 to 0.77 Hz (|*t*_12_| = −4.12, *p*<0.01) compared with the non-paretic hand.

In contrast, the analysis of the between-hand differences in the control group did not reveal a significant Hand×Frequency on the normalized power spectrum of force below 1 Hz (*p>*0.1; Figure 6C). Similarly, analyses of hand and frequency bands were not found significant (*p>*0.1; Figure 6D).

### Prediction of the Force Variability from Power below 1 Hz

**Figure 7:**
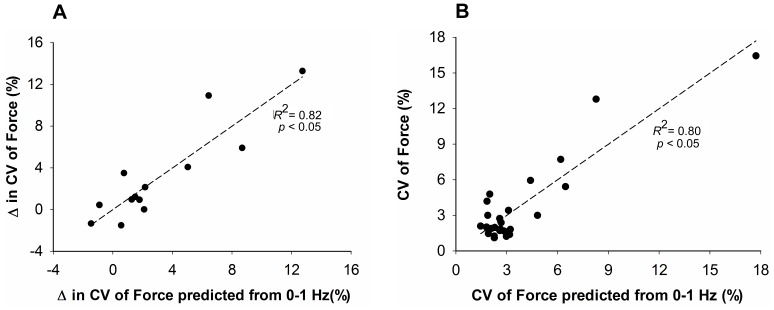
The prediction of CV of force from power below 1 Hz. *(A) For stroke and control group* ***.*** A backward multiple linear regression model was applied to predict CV of force (dependent variable) from the absolute power contributions at frequencies below 1 Hz (independent variable). The model predicted *(R^2^* = 0.82) the CV with specific contributions from frequency at 0.28 Hz (part *r* = 0.86) and 0.63 Hz (part *r* = −0.40). *(B) Within stroke group.* A backward multiple linear regression model was applied to predict the difference in CV of force (dependent variable) between hands in stroke participants from the differences in the power below 1 Hz (independent variable). The model predicted (*R* ^2^ = 0.80) the difference in CV of force with specific contributions from frequency at 0.35 Hz (part *r* = 0.82) and 0.63 Hz (part *r* = −0.32).

Next, we examined the extent to which the modulation of spectral power below 1 Hz in stroke and control participants was related to the variability of force. The CV of force was predicted using a backward multiple-regression model that included the absolute power at 0.28 Hz and 0.63 Hz (*R^2^* = 0.82, adjusted *R^2^* = 0.80; *p*<0.05; Figure 7A). This regression model indicated that increased CV of force was associated with greater absolute power at 0.28 Hz (part *r = *0.86) and lesser absolute power at 0.63 Hz (part *r* = −0.40).

The difference in the CV of force between the paretic and non-paretic hand of stroke participants was predicted by a backward multiple-regression model that included the absolute power at 0.35 Hz and 0.63 Hz (*R^2^* = 0.80, adjusted *R^2^* = 0.76; *p*<0.05; Figure 7B). This regression model revealed that an increased difference in CV of force was associated with greater absolute power at 0.35 Hz (part *r* = 0.82) and lesser absolute power at 0.63 Hz (part *r* = −0.32).

## Discussion

This study investigated the modulation of force below 1 Hz and its relation to increased force variability following stroke. Frequency specific changes in power below 1 Hz reflect a fundamental change in how the motor system functions after stroke. Our observations suggest that stroke increases force oscillations ∼0.2 Hz and reduces force oscillations ∼0.6 Hz compared with healthy adults. These results are consistent with previous findings that linked these specific frequencies to increased force variability in healthy young and older adults [1].

### Modulation of Force below 1 Hz and Force Variability

A major impairment following stroke is increased force variability, which affects the ability to control motor output. For example, we and others have previously shown that stroke individuals exhibit increased force variability relative to age-matched healthy adults during unimanual [6]–[8] and bimanual [5] force contractions. Greater force variability in stroke individuals is clinically relevant because of the association with the severity of stroke impairments (lower Fugl-Meyer scale score; [6]). In line with previous work, our findings demonstrate that force variability was greater in stroke individuals compared with healthy adults.

For the first time in the stroke literature, we investigated whether the modulation of force oscillations below 1 Hz is different between stroke individuals and healthy adults. The findings indicate that stroke individuals exhibited greater normalized power ∼ 0.2 Hz and lesser power ∼0.6 Hz compared with healthy adults (Figure 5B). Similarly, our results show that stroke individuals modulate force oscillations below 1 Hz differently with the paretic and non-paretic hand (Figure 6B). These differences are comparable to the differences in force modulation below 1 Hz between the paretic hand and non-dominant hand of healthy controls. In contrast, the healthy individuals exhibited no systematic differences in the modulation of force oscillations below 1 Hz with the dominant and non-dominant hands (Figure 6D). These findings are in line with our recent findings in young and older adults, which suggest force variability is associated with specific frequency oscillations below 1 Hz [1].

The most important finding in this study, however, was that the altered modulation of force below 1 Hz explained the difference in force variability between stroke individuals and healthy adults, and between the paretic and non-paretic hand. Parallel to the findings that compared young and elderly adults [1], we found that greater power at 0.28 Hz was associated with increased force variability, whereas greater power at 0.63 Hz was associated with reduced force variability. Therefore, our findings demonstrate the significance of the modulation of force below 1 Hz in stroke individuals. These findings provide further insight into identifying potential mechanisms underlying impaired force control following stroke.

### Possible Mechanisms

A major question that arises from the above findings is the following: *Why do stroke individuals exhibit greater power ∼0.2 Hz and reduced power ∼0.6 Hz compared with healthy adults?*

The amplified oscillations in force ∼0.2 Hz for stroke individuals may be due to the following: 1) An altered modulation in the discharge of motor units. Chou et al., 2013 report that rate coding is compressed and reduced in the paretic limb during constant force contractions [12]. Such altered motor neuron firing has been related to low-level excitatory synaptic input from spinal and supraspinal centers [14]. Further, Liang et al., (2010) demonstrated that stroke individuals exhibit longer after-hyperpolarization potential than age-matched healthy adults [13]. 2) Respiratory rhythm. Empirical evidence suggests that the respiratory capacity and rhythm is affected following stroke [15]. Normal breathing occurs at about 0.2 Hz and has been shown to be associated with force variability [22]. Whether respiratory rhythm has any direct influence on modulation of power ∼0.2 Hz in the force output remains a question for future investigation.

The reduced oscillations in force ∼0.6 Hz for stroke individuals may be because of impaired transformation of visual information into motor output (Figure 6B). Our previous findings indicate that in healthy young and older adults increased modulation of force at 0.6 Hz is associated with magnified visual feedback [1]. Furthermore, prior reports suggest that stroke individuals exhibit impairments in motor control with augmented visual feedback [16]. Although we did not manipulate visual feedback in the current study, perhaps the stroke individuals had difficulties in using the force feedback and transforming the feedback into a steady force output. Following stroke, the motor system is typically more impacted than the visual system. As seen in Figure 6B and D, stroke individuals exhibited similar modulation ∼0.6 Hz with the non-paretic hand compared with the dominant and non-dominant hands of the healthy subjects. Therefore, the decreased power at ∼0.6 Hz observed in the paretic hand of stroke individuals may be associated with an impaired ability to transform visual information into a motor output.

A number of physiological mechanisms can potentially explain the increased force variability in stroke individuals and possibly the altered modulation in force below 1 Hz. Most of the physiological mechanisms are derived from studies that compared young and older adults. The age-associated difference in force variability can be influenced by afferent input from the periphery, the firing properties of motor units innervating the muscles, as well as the descending drive on the spinal motor neurons [23]–[26]. Specifically, increased force variability in older adults is linked with greater variability in the discharge rate of motor units [27]. Further, force variability is influenced by the oscillatory input from the central drive originating from the interactions between cortical and subcortical structures such as primary and secondary motor areas, basal ganglia, thalamus and cerebellum [28], [29]. Future research should examine the relation between central and peripheral factors and force modulation around 0.2 and 0.6 Hz. In addition, the heterogeneity of stroke participants which include the lesion location and volume on modulation of force below 1 Hz may be an important consideration in the modulation of spectral power below 1 Hz.

In summary, our findings clearly demonstrate that the amplified force variability in stroke individuals is associated with a differential modulation of force below 1 Hz. Specifically, we show that stroke individuals exhibit increased power ∼0.2 Hz and reduced power ∼0.6 Hz compared with healthy adults. Therefore, the force oscillations below 1 Hz provide important information for understanding the amplified force variability and consequently pathological motor control following stroke.
